# Nutrient driven transcriptional changes during phage infection in an aquatic Gammaproteobacterium

**DOI:** 10.1111/1462-2920.15904

**Published:** 2022-01-26

**Authors:** Emelie Nilsson, Ke Li, Matthias Hoetzinger, Karin Holmfeldt

**Affiliations:** ^1^ Centre for Ecology and Evolution in Microbial Model Systems (EEMiS), Department of Biology and Environmental Science, Faculty of Health and Life Sciences Linnaeus University Kalmar SE‐39231 Sweden

## Abstract

Phages modulate bacterial metabolism during infection by regulating gene expression, which influences aquatic nutrient cycling. However, the effects of shifting nutrient regimes are less understood. Here, we analyzed transcriptomes of an ecologically relevant Gammaproteobacterium and its lytic phage in high (HNM) and low (LNM) nutrient medium. Despite different infection characteristics, including reduced burst size and longer latent period in LNM, the phage had a fixed expression profile. Bacterial transcription was instead different depending on nutrient regime, with HNM bacteria focusing on growth while LNM bacteria focused on motility and membrane transport. Additionally, phage infection had a larger effect on bacterial gene expression in LNM compared to HNM, e.g. suppressing increased iron uptake and altering expression of phosphorus uptake genes. Overall, phage infection influenced host metabolism more in LNM, which was more similar to natural conditions, emphasizing the importance of considering natural conditions to understand phage and host ecology.

## Introduction

Viruses that infect bacteria, phages for short, are major players in aquatic food webs. One of their most tangible effects is that they infect and kill approximately 20% of the bacterial community on a daily basis (Suttle, 1994, 2005). This incursion into the microbial loop (Azam *et al*., 1983) results in the lysis of bacteria, keeping the energy and nutrient flow within the microbial community instead of being transferred to organisms at higher trophic levels (Bratbak *et al*., 1990; Fuhrman, 2000). Besides killing their hosts, the infected bacterial community is first influenced by their phages. The infection is initiated when the phage encounters and attaches to its host, whereupon the phage DNA is transferred into the bacterium. After overcoming potential host defenses (Samson *et al*., 2013), the phage may have the capability to take over the host metabolic machinery. This can be seen as a crude redirection of the host machinery from producing bacterial building blocks to producing phage DNA and structural components, but it can also be a more complex manipulation of metabolic processes before bacterial lysis (Forterre, 2011), including the expression of phage auxiliary metabolic genes (Lindell *et al*., 2007; Chevallereau *et al*., 2016).

High‐throughput sequencing of mRNA has facilitated the study of these interactions by enabling sequencing of both host and phage transcripts during the course of infection. This has shown that phage genes are often expressed sequentially in either two or three temporally clustered modules (Lindell *et al*., 2007; Halleran *et al*., 2015; Howard‐Varona *et al*., 2017; Morimoto *et al*., 2018). The bacterial genes are commonly underexpressed during phage infection (Halleran *et al*., 2015; Doron *et al*., 2016; Leskinen *et al*., 2016). However, overexpression of particular bacterial genes has also been seen, either as a bacterial response to the infection or as the phage hijacks the bacterial machinery for its own need (Leskinen *et al*., 2016; Lin *et al*., 2016; Howard‐Varona *et al*., 2017). Despite differences in infection success, expression profiles of phage genes appear to be highly similar during infection, both when specific phages infect different hosts (Doron *et al*., 2016; Howard‐Varona *et al*., 2017) and when different phages infect the same host (Blasdel *et al*., 2017). Hence, differences in infection success appear to be driven by transcriptional changes of host genes (Doron *et al*., 2016; Howard‐Varona *et al*., 2017).

Recent research highlights the importance of understanding phage–host transcriptional interactions, especially in an ecological context (Clokie *et al*., 2020; Howard‐Varona *et al*., 2020; Zimmerman *et al*., 2020). However, transcriptomics experiments are usually performed in laboratory environments where the microorganisms are cultivated in high nutrient medium compared to environmental scenarios where nutrients might be scarce. Nutrient limitation has been shown to lead to failed or less successful phage infections (Moebus, 1996; Hadas *et al*., 1997), whereas nutrient addition to natural communities has been shown to lead to increased viral production (Motegi and Nagata, 2007). These changes in phage productivity caused by nutrient limitation point towards differences in gene expression. For bacteria alone, nutrient amendments have resulted in increased expression of genes coupled to metabolic pathways (McCarren *et al*., 2010; Poretsky *et al*., 2010; Beier *et al*., 2015; Lin *et al*., 2016). In phosphate limited experiments, both marine cyanobacteria and green algae altered their transcriptional expression to overexpression of genes involved in phosphate metabolism, while the influence of viral infection on the expression pattern was limited (Lin *et al*., 2016; Bachy *et al*., 2018). However, studies regarding the effect of phage infection combined with nutrient limitation on gene expression of heterotrophic aquatic bacteria have not yet been conducted and further investigations are needed to fully comprehend the dynamics of phage‐host systems during different nutrient regimes.

In this study, we investigated phage and bacterial growth dynamics as well as transcriptional response in two different nutrient concentrations with the model system *Rheinheimera* sp. BAL341 (hereafter BAL341) and Rheinheimera phage vB_RspM_Barba18A (hereafter barba18A). The host belongs to the Gammaproteobacteria class and is suggested to be ecologically relevant since it has shown metabolic plasticity when exposed to, e.g. transplant experiments (Lindh *et al*., 2015) and the organic pollutant alkane (Karlsson *et al*., 2019). The phage has a circular, dsDNA genome of 80 kb and shows a myovirus morphology (Nilsson *et al*., 2019). The bacterium and the phage were isolated at the Linnaeus Microbial Observatory in the Baltic Sea in July 2012 and August 2015 respectively. Both the host and the phage show temporal patterns with recurring peaks in abundance in the Baltic Sea during late summer (Nilsson *et al*., 2019). This coincides with the decay of cyanobacterial blooms (Bertos‐Fortis *et al*., 2016; Bunse *et al*., 2019), which indicates that BAL341 thrives during nutrient‐rich conditions and could be particle associated. We hypothesised that the phage would express its genes in a fixed manner independent of nutrient treatment, and that potential differences in replication would be driven by changes in host gene expression in the different nutrient treatments. The results showed that phage infection dynamics differed depending on nutrient concentration, but the expression of phage genes showed similar temporal clustering independent of nutrient treatment. The bacterial transcriptional response, however, was greatly affected by the nutrient treatment alone, and phage infection caused larger transcriptional changes of bacterial genes during low nutrient conditions. Overall, nutrient conditions are important variables when investigating phage transcriptional reprogramming of bacteria and their implications for nutrient cycling in ecological contexts.

## Results and discussion

### Infection characteristics

**Fig. 1:**
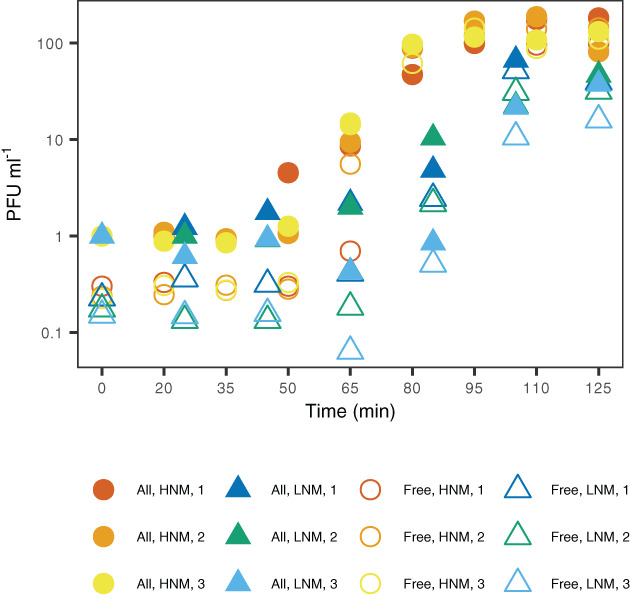
One‐step growth curves of barba18A infecting BAL341 in either HNM (high nutrient medium; circles in red, orange and yellow) or LNM (low nutrient medium; triangles in dark blue, green and light blue). *All phages* (filled symbols) indicate the total amount of phages at each time point, while *free phages* (open symbols) indicate phages that were free in the medium and not attached to or infecting bacteria. The one‐step growth curves were performed in triplicates for each treatment, and each replicate is shown. Plaque forming units (PFU) ml^−1^ are normalized against the total number of viruses at time zero for each replicate.

Bacteria were grown in two different media, high (HNM) and low (LNM) nutrient medium. HNM contained 242 mM carbon (Gómez‐Consarnau *et al*., 2007) and LNM contained 5% as much, 12.1 mM carbon. This is higher than compared to the Baltic Sea (0.33–0.47 mM dissolved organic carbon; Bunse *et al*., 2019), but in the range of what can be found in particles (potentially orders of magnitude higher than surrounding water; Prézelin and Alldredge, 1983; Herndl, 1988; Kaltenböck and Herndl, 1992). The nutrient levels resulted in a shorter generation time in HNM than LNM, based on bacterial growth curves calculated with colony‐forming units (CFU; generation time HNM: 87 ± 7 min, LNM: 142 ± 9 min; Supplementary Fig. 1). Furthermore, the number of bacteria (CFU) at a specific OD was higher in LNM compared to HNM, e.g. OD of 0.1 represented roughly 5.0 × 10^7^ CFU in HNM and 1.6 × 10^8^ CFU in LNM (Supplementary Fig. 2), suggesting a larger cell‐size for bacteria grown in HNM compared to LNM. One‐step growth curves were performed during bacterial exponential growth phase (OD ~0.1) by addition of phage at a multiplicity of infection (MOI) of 0.1. The one‐step growth curves showed that the phages that infected bacteria grown in HNM had a shorter latent period and larger burst size (55 ± 9 min and 169 ± 17 PFU produced per infecting phage respectively, *n* = 3) compared to the phages that infected bacteria grown in LNM (65 ± 0 min and 34 ± 13 PFU produced per infecting phage respectively, *n* = 3; Fig. 1). Phage adsorption curves showed that more than 80% of the phages had adsorbed to the bacterial hosts in both treatments within 7.5 min after addition (Supplementary Fig. 3), which coheres with the percentage of infecting phages based on time point 0 in the one‐step growth curves (5 min after phage addition; HNM: 75 ± 4%, LNM: 82 ± 4%; Fig. 1). This ensures synchronized infection patterns with the majority of bacteria within each sample being infected within a short time frame.

### Transcriptional takeover by phage genes

**Fig. 2:**
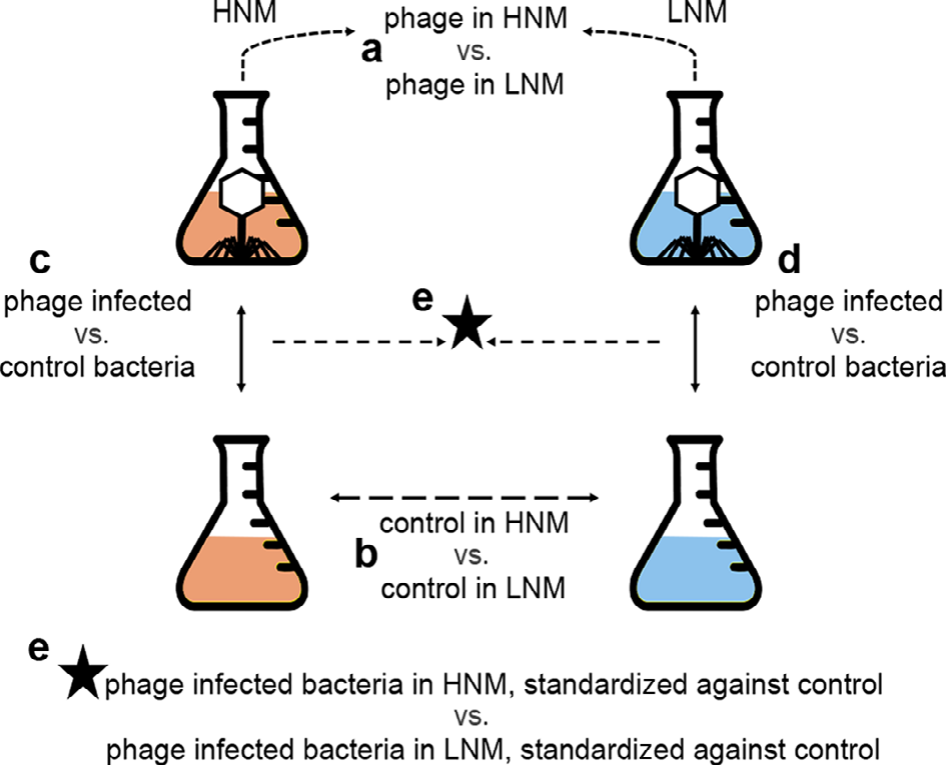
Explanation of experimental setup and how the different treatments were compared. Each treatment was replicated three times. Phages depicted within the flasks indicate the infected treatments while the colors [reddish for high nutrient medium (HNM) and bluish for low nutrient medium (LNM)] indicate nutrient treatments. The arrows indicate which treatments were compared during the different differential expression analyses, where the letters a–e facilitate the references to this figure in the main text.

**Fig. 3:**
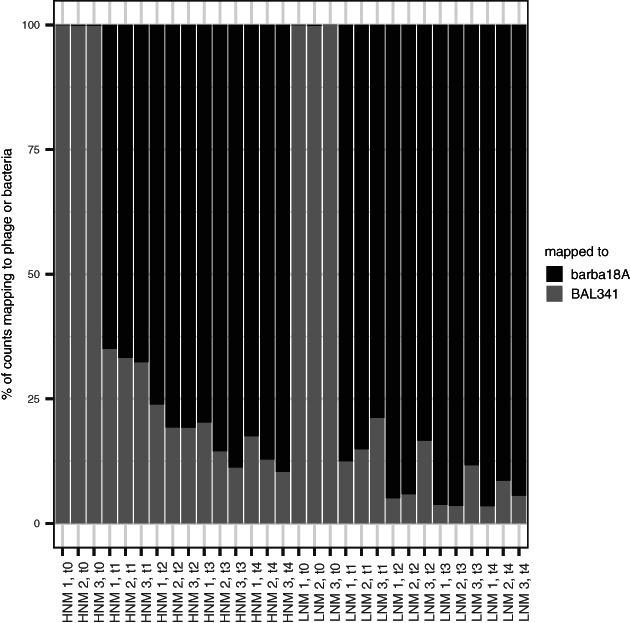
Percentage of reads mapped to either the phage (barba18A) or bacterial (BAL341) genome for phage‐infected samples. The first 15 samples are from the high nutrient medium (HNM) treatment and the following 15 are from the low nutrient medium (LNM) treatment.

Transcriptomic experiments were performed similarly as for the one‐step growth curves, except with a higher MOI (2.5 or 8 instead of 0.1), making sure as many cells as possible were infected at the same time. These were carried out in HNM and LNM, and each condition had a control treatment where water was added instead of phage (Fig. 2). Samples for RNA were taken at five different time points during the latent period, representing 1 min after the addition of phages and approximately 25%, 50%, 75% and 100% of the latent period. For phage treatments, the number of reads from each library that mapped to either the phage or bacterial genome changed during the course of the experiment in both HNM and LNM in a similar manner (Fig. 3, Supplementary Table 1). One minute after infection (time point 0), only a limited number of reads (0.1%–0.3%) mapped to the phage genome (Supplementary Table 1). These reads were not concentrated to any specific phage region but distributed across the phage genome in the same manner as reads from the control treatments. This implies that the phage had not taken over the host machinery to express its own genes and only host gene expression was ongoing. However, at time point 1, 65%–87% of the reads mapped to the phage genome, which increased to a maximum of 96% at later time points (Fig. 3). Such transcriptional takeover has been seen in various phage‐host systems (Halleran *et al*., 2015; Doron *et al*., 2016; Blasdel *et al*., 2017), while other phage genomes are expressed at much lower levels (15%–30% phage reads) during infection (Leskinen *et al*., 2016; Morimoto *et al*., 2018). This indicates that barba18A efficiently redirected the transcription machinery towards expression of phage genes, thereby replacing host gene expression over time and enabling phage propagation.

### Phage transcription

**Fig. 4:**
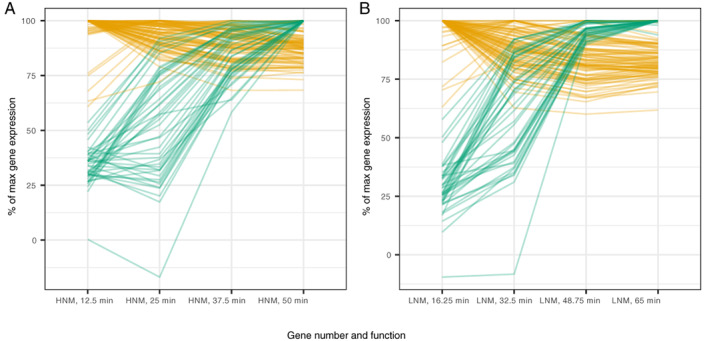
Phage gene expression over time in the different treatments. Clustering of genes in (A) high nutrient medium (HNM) and (B) low nutrient medium (LNM). The clustered genes that have yellow lines are genes that have high transcript levels from the start and then are kept at high levels throughout the infection cycle (i.e. early genes), while green lines are genes that have initial low transcript levels and increase towards the end of the infection cycle (i.e. late genes). The data are based on edgeR's average cpm‐function (samples with more data are given more weight) for the triplicates at each time point and normalized so that the maximum expression of each gene is set to 100% and the other transcript levels are shown as percentage of the maximum. Information for the individual genes can be found in Supplementary Table 2.

Independent of nutrient treatment, the phage genes were transcribed in two temporal clusters depending on when each gene had its maximal relative expression (Fig. 4, Supplementary Table 2). Genes in the first group (yellow) peaked at time point 1 (i.e. 12.5 and 16.25 min in HNM and LNM respectively) and 2 (i.e. 25 and 32.5 min in HNM and LNM respectively) and were thereafter maintained at relatively high expression levels throughout the experiment (>60%; Fig. 4). The second group (green) started at low levels of relative expression (<60%), then increased during time point 2 or 3, and reached their maximal relative expression at time point 4 (Fig. 4). The first group of genes mainly coded for proteins involved in genome replication and nucleotide metabolism, while the second group of genes mainly coded for structural proteins as well as proteins involved in virion assembly and DNA packaging. Commonly, phage genes are expressed in three temporal clusters, with an early group of host takeover genes, a subsequent group involved in genome replication, and a late group that produces the mature virion (Yoshida‐Takashima *et al*., 2012; Halleran *et al*., 2015; Morimoto *et al*., 2018). Here, we only detected the two later clusters, and we were not able to distinguish any genes involved in host takeover. This could be due to the unfortunate timing of our sampling, since there was no phage gene expression at time point 0 and the signal of potential host takeover genes was mixed with middle genes at time point 1.

The phage genes in both treatments were clustered in the same groups (Supplementary Table 2), indicating that the phage expression was similar at similar times and not at specific portions of the latent period. Looking at differentially expressed (DE) phage genes at the same proportions of the latent period also supported this. At 25% of the latent period there was a large number of underexpressed genes in HNM within the first cluster followed by underexpressed genes in HNM within the second cluster at 50% and 75% of the latent period [overexpression is when log2‐fold change (logFC) >0 and underexpression is when logFC <0]. These shifts are likely driven by the fact that LNM was sampled at later time points and thus would have produced a larger number of mRNA transcripts (Supplementary Table 3 columns D–K). Therefore, gene expression between nutrient treatments was compared at similar time points instead of percentage of latent period (12.5 vs. 16.25, 37.5 vs. 32.5 and 50 vs. 48.75 min in HNM and LNM: comparisons a, b and e in Fig. 2). In these comparisons (Supplementary Table 3 columns L–Q), DE phage genes in cluster one were underexpressed in HNM at the first compared time (12.5 vs. 16.25 min), likely due to LNM having had longer time to produce transcripts. At the later compared time (37.5 vs. 32.5 min), where HNM was sampled later, genes within cluster two were instead overexpressed in HNM, while genes in cluster one were underexpressed. At the last compared times, when the time difference in sampling was the smallest (50 vs. 48.75 min), there was only one DE gene, indicating that the time differences in sampling indeed affected the results.

The uniformity of phage gene expression clustering in both treatments is similar to previous results, where limited differences in phage temporal expression patterns were seen despite replication differences when infecting different hosts (Doron *et al*., 2016; Blasdel *et al*., 2017; Howard‐Varona *et al*., 2017). Thus, our findings extend the assumption of a fixed strategy for phage gene expression and suggest that other factors, e.g. host transcriptional capacity, mainly drive differences in replication efficiency, even for very different nutrient regimes.

### Bacterial expression depending on nutrient treatment

**Fig. 5:**
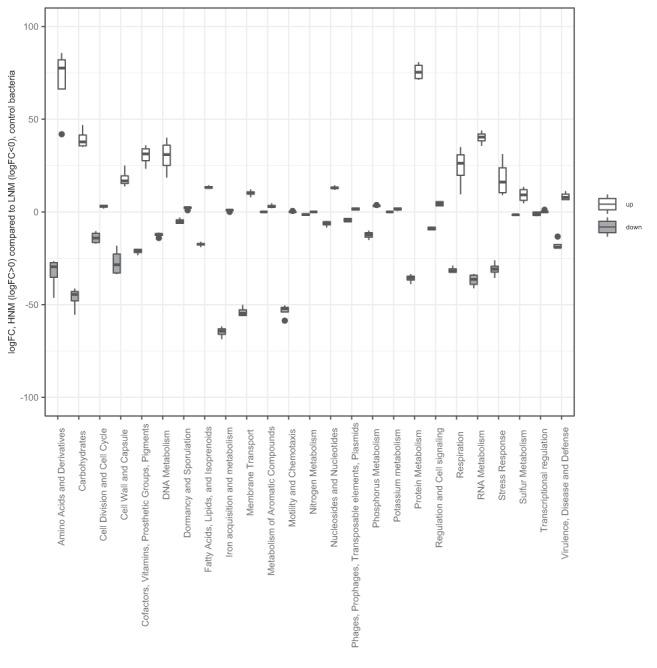
Differentially expressed (DE) genes in high nutrient medium (HNM) control bacteria compared to low nutrient medium (LNM) control bacteria. Total logFC for DE genes in each functional category was summarized per time point, which resulted in five different logFC, one for each time point, per category that is presented in the box plot (*n* = 5). Lower and upper box limits denote the 25th and 75th quartiles, thick line denotes the 50th percentile (median). The whiskers represent 1.5× interquartile range from the lower and upper quartiles, not encompassing extreme values (outliers) which are dots outside of this range. Information for the individual genes can be found in Supplementary Table 4.

The general transcript levels of bacterial genes differed between control bacteria in HNM compared to LNM (comparison b in Fig. 2), as well as between infected and non‐infected bacteria in both treatments (comparison c and d in Fig. 2, Supplementary Fig. 4). When comparing control (non‐infected) bacteria in the different nutrient treatments, 2096 of 3737 genes showed DE at any time point and 58% of those showed DE at three time points or more. Of these, 1133 genes were underexpressed in HNM compared to LNM, 962 genes were overexpressed and one gene showed both under‐ and overexpression depending on time point (Supplementary Table 4). Still, there was a relatively equal distribution of both under‐ and overexpressed genes within each functional category, with a few exceptions (Fig. 5). For example, genes involved in motility and chemotaxis, as well as membrane transport, were overall underexpressed in HNM compared to LNM. The overexpression of genes within these functional categories has previously been seen among carbon starved marine bacteria (Manck *et al*., 2020), and thus the results are expected as the bacteria in LNM were more limited in terms of carbon compared to HNM.

### Bacterial expression depending on infection

**Fig. 6:**
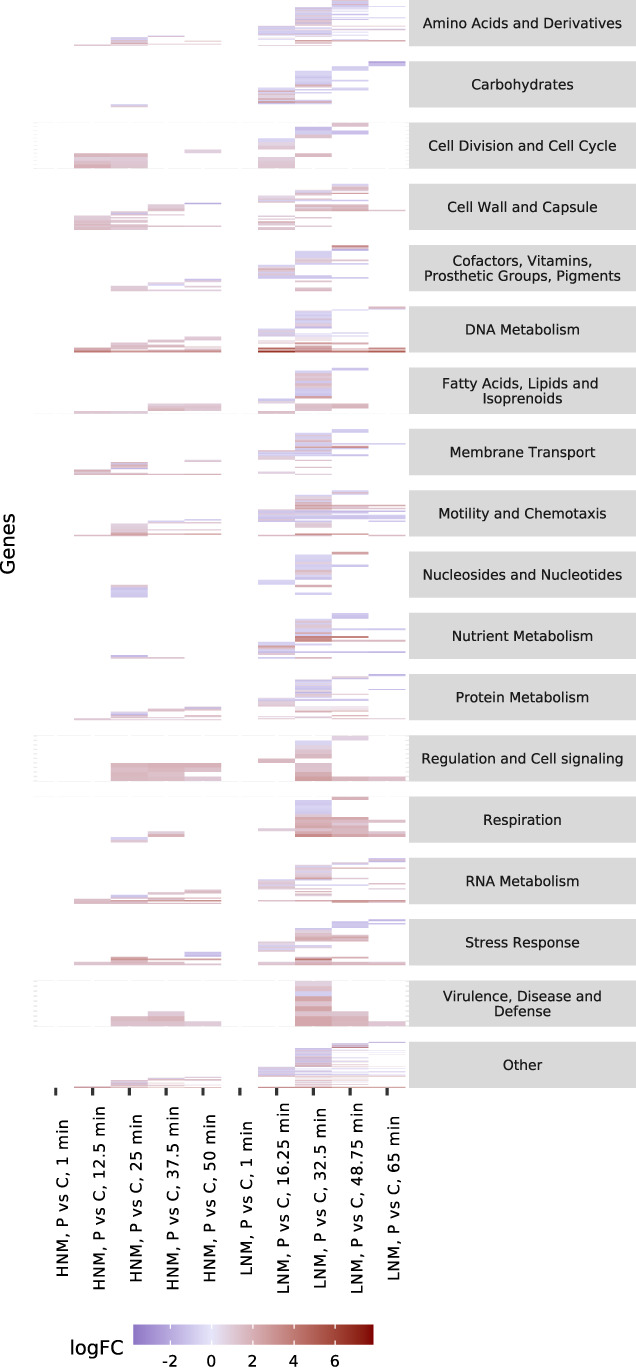
Differentially expressed (DE) genes between phage infected bacteria and control bacteria in each medium at each time point, organized based on functional annotations of genes. DE genes with logFC >0 are overexpressed in phage infected bacteria while DE genes with logFC <0 are underexpressed. The number of DE genes in each category varied accordingly: Amino Acids and Derivatives 61, Carbohydrates 30, Cell Division and Cell Cycle 12, Cell Wall and Capsule 32, Cofactors, Vitamins, Prosthetic Groups, Pigments 26, DNA Metabolism 46, Fatty Acids, Lipids, and Isoprenoids 18, Membrane Transport 39, Motility and Chemotaxis 32, Nucleosides and Nucleotides 18, Nutrient Metabolism 24, Protein Metabolism 47, Regulation and Cell signaling 10, Respiration 16, RNA Metabolism 35, Stress Response 27, Virulence, Disease and Defense 9, Other 445. Information for the individual genes can be found in Supplementary Table 5.

There were no DE genes when comparing phage infected bacteria and control bacteria at time point 0 in both HNM and LNM, emphasizing that the transcriptional response to infection had not started yet. At later time points, bacterial genes in phage infected cultures were generally overexpressed rather than underexpressed in both HNM and LNM (comparison c and d in Fig. 2, Supplementary Fig. 5) and the effect, both in the number of DE genes and the overall logFC, was larger in LNM compared to HNM (Fig. 6).

In HNM, 49 bacterial genes were underexpressed and 202 genes were overexpressed. In LNM, 360 genes were underexpressed and 469 were overexpressed. The two treatments shared 141 overexpressed genes, including genes for DNA replication [e.g. DNA helicase (VHO02543.1), DNA polymerase (VHO05078.1) and DNA repair protein (VHO05537.1); Supplementary Table 5] that could be involved in phage progeny production, or alternatively used by the host to repair degraded DNA. Furthermore, seven of the most highly overexpressed genes are likely involved in host defense. They are consecutively and coherently expressed and situated after each other on the bacterial genome (Supplementary Table 5). Four of them have unknown functions (VHO00353.1, VHO00355.1, VHO00361.1, VHO00363.1), while the other three are involved in restriction modification systems. These systems are known bacterial defense systems (reviewed in; Labrie *et al*., 2010) and these genes constitute two subunits for a site‐specific restriction enzyme (VHO00351.1 and VHO00357.1) and the associated methyltransferase (VHO00359.1). Another response to the infection was seen in overexpression of phage shock proteins A, B and C (VHO05102.1, VHO05101.1, and VHO05100.1; Supplementary Table 5) when comparing both phage treatments to their controls. These genes are known to be expressed as a reaction to phage infection (Brissette *et al*., 1990; Brissette *et al*., 1991). The overexpression of these genes in both phage treatments indicates their importance for either the host or the phage during infection.

Opposite to the overlap of most of the DE genes in phage infected HNM with DE genes in phage infected LNM, the LNM treatment had a large proportion (82%) of unique DE genes. Many of the underexpressed genes (70%) during phage infection in LNM were also DE when comparing HNM control bacteria to LNM control bacteria. However, in the latter comparison, these genes had a higher relative expression in LNM compared to HNM, indicating that the genes of importance for the non‐infected host in LNM were suppressed during phage infection. This implies that the transcriptional state of the bacteria growing in LNM was less optimal for the phage than in HNM. This is peculiar as the nutritional state in LNM more resembles the natural state, and the phage is likely more adapted to these circumstances. Instead, the bacterial transcriptional state in the unnatural HNM leads to a weaker transcriptional response to phage infection. In HNM, the host is focusing on functions that also benefits phage production, while in LNM, the host's focus on scavenging nutrients is of less interest for phage production. The process of refocusing the host to the phage's needs might have contributed to the longer latent period and smaller burst size in LNM, which then might be more similar to phage production in nature.

### Infection status and nutritional effect on iron

When comparing phage infected and non‐infected bacteria, five of the six bacterial DE genes associated with iron acquisition and metabolism were underexpressed in LNM (comparison d in Fig. 2), while no iron associated genes were DE in HNM (comparison c in Fig. 2, Supplementary Table 5). This contradicts previous studies that have shown increased transcript levels of iron associated genes during phage infection, potentially as a stress response (Blasdel *et al*., 2017; Sacher *et al*., 2018). When comparing only the control bacteria in HNM with LNM (comparison b in Fig. 2, Supplementary Table 4), 15 out of the 17 DE genes associated with iron were consecutively underexpressed in HNM compared to LNM. These mainly included various iron transporters, indicating an increased focus on iron acquisition in LNM. Of the overexpressed genes in LNM, two genes were underexpressed during phage infection: a ferric iron ABC‐transporter and an iron siderophore sensor protein (comparison d in Fig. 2, Supplementary Table 5), where the latter was consecutively underexpressed at the three last compared time points. Iron siderophores are important competing factors for aquatic bacteria (Eickhoff and Bassler, 2020) and can be utilized to gain access to host cells by phages as described in the ‘Ferrojan Horse Hypothesis’ (Bonnain *et al*., 2016). According to this hypothesis, the increased siderophore expression in LNM could leave the bacteria more vulnerable to phage infections. Therefore, suppression of these genes by the phage could prevent further phage infections and thereby increase the phage's fitness in nature.

### Changed expression of phosphorus related genes

Regarding genes involved in phosphorus metabolism, there was an overall underexpression in HNM control bacteria compared to LNM (comparison b in Fig. 2, Fig. 5, Supplementary Table 4) and the most underexpressed gene was the phosphate‐starvation inducible protein PhoH. PhoH has been suggested to be involved in phosphate metabolism (Kim *et al*., 1993) and has shown overexpression in bacteria during phosphate starvation (Wanner, 1993; Ishige *et al*., 2003; Poranen *et al*., 2006) and late stages of phage infection (Lindell *et al*., 2007). When comparing phage infected bacteria to their controls, no phosphorus‐related genes were DE for HNM while eight were DE in LNM (comparisons c and d in Fig. 2, Supplementary Table 5). Of these eight genes, three were overexpressed and five underexpressed. Two of the underexpressed genes in phage infected LNM were overexpressed in the LNM control compared to HNM: an alkaline phosphatase and *phoH*. The higher expression in LNM control bacteria compared to HNM control bacteria might be an indication of an increased phosphorus demand in this low nutrient medium, while the reduced expression in LNM during infection is more difficult to explain. Potentially, the suppression of host *phoH* could be a counteractive response caused by the expression of the phage's own *phoH* gene (Supplementary Table 2).

Of the three phosphorus‐related genes that were overexpressed in phage infected LNM bacteria, the bacterial phosphate ABC transporter *pstS* was highly overexpressed at several time points (Supplementary Table 5). This gene was also overexpressed in LNM relative to HNM when comparing phage infected bacteria, standardized against the controls, in the different nutrient treatments (comparison e in Fig. 2, Supplementary Table 6). The *pstS* gene is part of the pho regulon and is known to increase uptake of phosphorus during depleted conditions (Wanner, 1993). Enriched transcript levels during phosphorus starvation and phage infection have previously been seen, even when the phage carried its own *pstS* gene (Lin *et al*., 2016). Thus, *pstS* gene overexpression is likely essential to produce further phosphate‐rich phage progeny in phosphate limited environments. This highlights how the phage is able to hijack and reroute the host's metabolism to suit its own needs, emphasizing the importance of considering phage infections at nutrient concentrations mimicking natural systems.

## Conclusions

To fully acknowledge the impact of bacteria on nutrient and carbon cycling in natural environments, we need to understand the impact of phages on bacterial metabolism during infection (Howard‐Varona *et al*., 2020; Zimmerman *et al*., 2020), especially considering that 20% of the bacterial community is assumed to be infected on a daily basis (Suttle, 2005). Here, we show that different nutrient conditions during phage infection led to a different transcriptional response in the host but not the phage despite differences in infection success. In our HNM treatment, which is nutrient enriched compared to Baltic Sea conditions, we have a shorter latent period and larger burst size than in our LNM treatment, but a relatively small host transcriptional response caused by the phage infection. The infection in LNM, which simulates natural conditions to a larger extent, brought on a larger transcriptional response due to phage infection with a larger number of both under‐ and overexpressed genes. Particularly the transcriptional changes for both iron and phosphorus acquisition genes are highly relevant in an ecological context with regards to potential impact on nutrient cycling. The implications of this are obvious as natural environments are heterogeneous and current micro‐scale conditions will influence the course of the phage infection. While this is the first study investigating expression profiles of phage infected heterotrophic bacteria under different nutrient conditions, and further research will be necessary to reveal if this behavior is general among other aquatic phage‐host systems, the results are likely not unique to this system and add another layer of complexity to the relationship between bacteria and phages. Considering how our environment is changing, it is vital to understand what shapes current ecological processes to be able to estimate future scenarios and understanding how infected and non‐infected bacteria behave is an important part of the puzzle.

## Experimental procedures

### Bacterial strain, phage and growth conditions

The bacterial strain used in these experiments was *Rheinheimera* sp. BAL341 (CAAJGR010000000) and the phage was Rheinheimera phage vB_RspM_Barba18A (MK719729). The first treatment, HNM (242 mM carbon; Gómez‐Consarnau *et al*., 2007), consisted of standard Zobell medium [1 g yeast extract (Becton, Dickinson and Company (BD), Franklin Lakes, NJ, USA) and 5 g bacto‐peptone (BD) in 800 ml filtered (Whatman glass microfiber filter, GF/C, GE Healthcare, Chicago, IL, USA) Baltic Sea water and 200 ml Milli‐Q water: this, and all other media, was autoclaved at 121°C for 20 min]. The second treatment, LNM (12.1 mM carbon), consisted of a 5% Zobell solution, where the HNM was diluted in filtered Baltic Sea water. BAL341 was incubated at room temperature (RT) with agitation. Bacterial growth experiments were performed in triplicates for both HNM and LNM by inoculating one colony of BAL341 into 10 ml of each medium. Optical density (OD_600_) and CFU samples were extracted at different time points for the two media. For HNM, the measurements were taken every hour during the first 9 h, and then measured two additional times for a total of 12 h. For LNM, the measurements were taken every second hour for 21 h in total. Optical density was measured with Biowave CO8000 Cell Density Meter. Bacteria for CFU enumeration were spread on Zobell‐agar plates [Zobell medium with 15 g bacto agar (BD) per litre], the plates were incubated at RT, and colony formation was monitored for 48 h. For each phage experiment, the bacteria were grown to an OD_600_ of roughly 0.1. To achieve this for HNM, 500 μl of an overnight culture was transferred to 10 ml fresh medium and was thereafter grown until the desired OD_600_. In LNM, a bacterial colony was inoculated in LNM and left to grow overnight and during the following day until the desired OD_600_ was reached.

Adsorption analysis was performed to investigate how fast the phage attached to the host. Bacteria grown in HNM or LNM and phages were mixed with an MOI of ~0.1. After 1 min, the first sample was collected and filtered through a 0.22 μm filter (Merck Millipore, Darmstadt, Germany) and subsequent samples were collected every 2.5 min for 15 min. The filtrate, which contained free phages that had not adsorbed to the host, was enumerated through plaque assays. On a Zobell‐agar plate, 300 μl overnight culture of BAL341 and 100 μl of barba18A filtrate were mixed together with 3.5 ml of top‐agar [marine sodium magnesium buffer: 450 mM NaCl (Sigma, St Louis, MO, USA), 50 mM MgSO4 × 7H_2_O (Merck Millipore) and 50 mM Trizma base (Sigma), pH 8; with 0.5% low melting point agarose (Thermo Fisher Scientific, Waltham, MA, USA)]. Plates were incubated at RT and plaque‐forming units were monitored for 48 h.

One‐step growth curves were performed for barba18A with BAL341 in HNM as described in Nilsson *et al*. (2019), and performed similarly in LNM, but with slight modifications. Briefly, both cultures were grown to an approximate density of 10^8^ cells ml^−1^. This was measured with OD at the time of sampling and calculated based on previously established correlations between OD and CFU ml^−1^ (Supplementary Fig. 2). Thereafter, phages were added to a final concentration of 10^7^ phages ml^−1^ (MOI 0.1) and left to incubate for 5 min before they were diluted 1000‐fold to reduce the number of new infections. Samples for enumeration of free and total phages were withdrawn directly after the dilution, and thereafter every 15th minute for HNM and every 20th minute for LNM for 2 h in total. For total phages, which include both bacterial‐attached and free phages, samples were retrieved directly from the phage infected bacterial culture, while the samples for free phages were filtered through a 0.22 μm filter (Merck Millipore). The samples were enumerated with plaque assays as described above, and plaque formation was monitored for 48 h. The increase in phages indicated when the burst took place and allowed for determination of the latent period, which was calculated as the average of the time until both all and free phages increased for each replicate. Burst size was calculated as the difference between the number of free viral particles after the burst and the number of free viral particles before the burst divided by the number of viruses that had infected a host. The number of viruses that had infected a host was calculated as the difference between total number of viruses before the burst minus the number of free viruses before the burst. All viral abundances were normalized against the total number of viruses at time zero for each replicate.

### RNA experiment set‐up and sampling

To gather samples for RNA extraction, bacteria were grown as previously described in both LNM and HNM and pooled into two sets of 50 ml for each nutrient concentration. These experiments were done with a larger volume and different MOI than the one‐step growth curves described above, but the same infection dynamics were assumed. To one bottle of each nutrient concentration, barba18A was added at an MOI of either 2.5 or 8 (for LNM and HNM respectively, based on CFU at OD 0.1; 104.6 μl), and to the other bottle, the control, an equal volume of MilliQ water was added. Samples for RNA extraction were collected at five time points during the infection cycle, including 1 min after addition of phages and then at approximately 25%, 50%, 75% and 100% of the latent period to encompass all phases of the infection. Latent periods were calculated from the one‐step growth curves described above. Thus, RNA was collected for HNM at 1, 12.5, 25, 37.5 and 50 min, and for LNM at 1, 16.25, 32.5, 48.75 and 65 min. Triplicate experiments were conducted for both HNM and LNM (Fig. 2). Originally, sampling at 175% of the latent period was included, but this was excluded from the results and discussion as the majority of cells had lysed in phage treatments and the remaining cells were subjected to non‐synchronized infections.

At each time point, three 1 ml samples of each treatment were added to 500 μl RNA protect (Qiagen, Venlo, Netherlands), vortexed for 5 s, incubated for 5 min before 10 min centrifugation at 5000*g*. After the supernatant was removed, the samples were flash‐frozen in liquid nitrogen and stored at −80°C until extraction.

RNA extractions from all samples were performed with RNeasy Mini kit (Qiagen) following the manufacturer's protocol and eluted in pre‐heated (50°C) nuclease‐free water. The samples were then DNase treated with the Ambion Turbo DNA free kit (Invitrogen by Thermo Fisher Scientific, Carlsbad, CA, USA) according to the manufacturer's protocol to remove any DNA. Potential remaining DNA was estimated with PCR by amplifying the bacterial 16S region as described in Holmfeldt *et al*. (2007). The two samples still containing DNA were DNase treated a second time. Thereafter, all samples were rRNA depleted with the RiboMinus transcriptome isolation kit and the RiboMinus concentration module (Invitrogen), according to the manufacturer's recommendations. The resulting RNA was eluted in nuclease‐free water.

### cDNA synthesis and sequencing

Both cDNA synthesis and sequencing were performed by SciLife/NGI (Solna, Sweden). Illumina TruSeq Stranded mRNA kit (Illumina, San Diego, CA, USA) was used to convert the rRNA‐depleted RNA samples to cDNA and prepare the libraries. The libraries were then sequenced on a NovaSeq6000 (Illumina), resulting in pair‐end 50 bp long reads.

### Sequencing read processing

The workflow for processing the raw reads was organized with Snakemake (version 5.4.4, Supplementary Information 1) (Köster and Rahmann, 2012). First, reads were trimmed with regards to quality and adapter removal with Trimmomatic (version 0.36, settings: PE, ‐phred33 ‐illuminaclip:TruSeq2‐PE.fa:2:30:10 leading:3 trailing:3 slidingwindow:4:15 minlen:30) (Bolger *et al*., 2014). The quality of the reads was assessed with FastQC (version 0.11.6) (Andrews, 2010) and summarized with MultiQC (version 1.3) (Ewels *et al*., 2016). The trimmed reads were aligned to the genome of BAL341 and barba18A using bowtie2 (version 2.2.3) (Langmead and Salzberg, 2012) and sorted with samtools (version 1.6) (Li *et al*., 2009). The read alignments were summarized with featureCounts (version 1.6.2, settings: ‐p ‐B ‐C ‐T 1 ‐a ‐t CDS ‐g gene_id ‐d 45 ‐o) (Liao *et al*., 2013) and a count table was generated for downstream analysis in R.

### Statistical analysis of transcriptomic data

Statistical analysis and graphical visualization (with ggplot2, version 3.3.2) (Wickham, 2016) were performed in R (version 3.6.2) (R Core Team, 2018) through RStudio (version 1.2.5033, Supplementary Information 2) (RStudio Team, 2015). Reads that mapped to the bacterium and those that mapped to the phage were normalized separately (Supplementary Information 2), and the normalization was done with the trimmed mean of M values (TMM) normalization from the edgeR package (version 3.28.0) (Robinson *et al*., 2010).

To describe phage gene expression across time, the first time point (time 0), 1 min after phage addition, was excluded since the amount of phage gene expression was very low (0.06%–0.26%) and did not represent actual phage infection. Including those samples would have inflated the normalization. Temporal clustering was performed on TMM normalized counts that were transformed with edgeR's average log counts per million function (aveLogCPM). These counts were further normalized for each gene by setting the maximum expression to 100% and the remaining time points were normalized against the maximum (Leskinen *et al*., 2016). Genes were clustered into groups based on when they had their maximum value.

To compare the expression of genes between treatments, we used the amount of mRNA (number of reads) as a proxy for gene expression. These are only relative numbers and not absolute values. The mRNA was therefore TMM normalized as mentioned above, and genes were termed DE when the TMM normalized counts were significantly different (FDR < 0.05) between the two treatments. DE genes were calculated between phage genes in HNM and LNM at each time point, between control bacteria in each medium and at each time point, between phage infected bacteria and control bacteria in each medium and at each time point, and between the phage infected bacteria in the two different media after standardizing against their controls (Fig. 2). Bacterial genes were categorized through RAST (Aziz *et al*., 2008) into RAST subsystem categories based on their functional annotations.

## Data Availability

Raw reads from the transcriptional experiment were deposited in NCBI under BioProject accession number PRJNA644242. Within this project, samples associated with the high nutrient medium were designated ‘full’ instead of ‘high’.

## Author Contributions

E.N. and K.H. designed the study; E.N. and K.L. carried out experimental procedures; E.N., K.L., M.H. and K.H. performed data analyses and interpretation; E.N. and K.H. wrote the initial manuscript with help and comments by K.L. and M.H.

## Supporting information


**Supplementary Information 1.** Instructions for how to run the Snakemake‐pipeline to process the sequencing reads.

**Supplementary Information 2.** Analyses performed in R on the counted reads to identify differentially expressed genes and produce figures.

**Supplementary Fig. 1.** Growth curves of BAL341 in high nutrient medium (HNM: High 1–3) and low nutrient medium (LNM: Low 1–3) based on colony‐forming units (CFU) ml^−1^.
**Supplementary Fig. 2**. Colony‐forming units (CFU) per ml plotted against OD measurements for bacteria in HNM and LNM, data collected from bacteria in exponential phase.
**Supplementary Fig. 3**. Adsorption of barba18A phages to BAL341 when mixed at an MOI of 0.1, performed in triplicates in HNM and LNM. Enumeration of the free phages in the mixture was used to calculate plaque‐forming units. The initial (0 min) concentration of phages was 1 × 10^7^ PFU ml^−1^.
**Supplementary Fig. 4**. MDS plot of the mRNA counts associated to the different samples. Orange circles are samples in the HNM treatment while blue circles are samples in the LNM treatment, which are clearly separated. Closed circles are phage infected bacteria while open circles are non‐infected bacteria. For those, phage infected samples at time zero overlap with non‐infected samples, while later samples are separated from the uninfected control.
**Supplementary Fig. 5**. Overall over‐ or underexpression (total log2‐fold change) of differentially expressed genes for the different samples when comparing phage infected cells (P) to control (C) cells.

**Supplementary Table 1.** General characteristics of the samples.

**Supplementary Table 2.** Phage gene expression in the two treatments (HNM and LNM) across the four analysed time points.

**Supplementary Table 3.** Phage differential expressed genes when comparing the two nutrient treatments (HNM and LNM).

**Supplementary Table 4.** Differentially expressed (DE) genes when comparing control bacteria in HNM with control bacteria in LNM.

**Supplementary Table 5.** Differentially expressed (DE) genes when comparing phage infected bacteria versus control within nutrient treatment.

**Supplementary Table 6.** Comparisons between phage infected bacteria, standardized against the controls, depending on nutrient treatment.
