# Supplementary material for: Nutrient driven transcriptional changes during phage infection in an aquatic Gammaproteobacterium
Source: Environ Microbiol. 2022 Jan 26;24(5):2270–81. doi: 10.1111/1462-2920.15904 (PMC9305737; doi:10.1111/1462-2920.15904)
Supplement: Supplementary file 3 — Supplementary Fig. 1. Growth curves of BAL341 in high nutrient medium (HNM: High 1–3) and low nutrient medium (LNM: Low 1–3) based on colony‐forming units (CFU) ml−1. Supplementary Fig. 2. Colony‐forming units (CFU) per ml plotted against OD measurements for bacteria in HNM and LNM, data collected from bacteria in exponential phase. Supplementary Fig. 3. Adsorption of barba18A phages to BAL341 when mixed at an MOI of 0.1, performed in triplicates in HNM and LNM. Enumeration of the free phages in the mixture was used to calculate plaque‐forming units. The initial (0 min) concentration of phages was 1 × 10^7^ PFU ml−1. Supplementary Fig. 4. MDS plot of the mRNA counts associated to the different samples. Orange circles are samples in the HNM treatment while blue circles are samples in the LNM treatment, which are clearly separated. Closed circles are phage infected bacteria while open circles are non‐infected bacteria. For those, phage infected samples at time zero overlap with non‐infected samples, while later samples are separated from the uninfected control. Supplementary Fig. 5. Overall over‐ or underexpression (total log2‐fold change) of differentially expressed genes for the different samples when comparing phage infected cells (P) to control (C) cells. [file EMI-24-2270-s007.pdf]

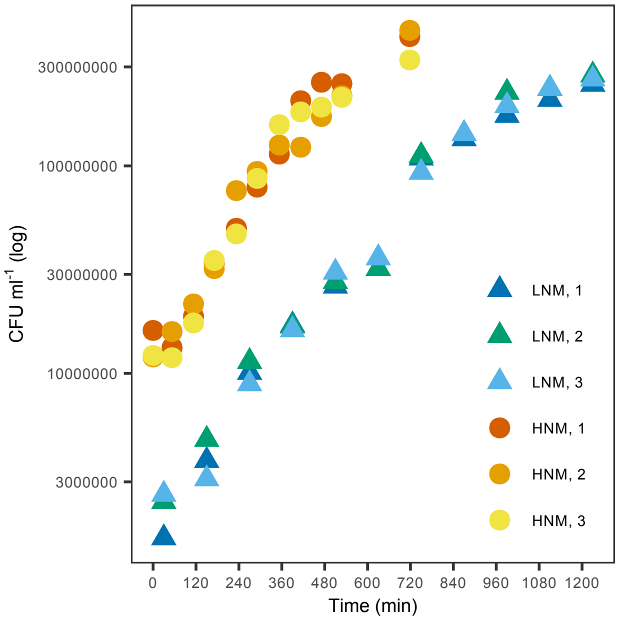

Supplementary figure 1. Growth curves of BAL341 in high nutrient medium (HNM: High 1-3) and low nutrient medium (LNM: Low 1-3) based on colony forming units (CFU) ml^{-1}.

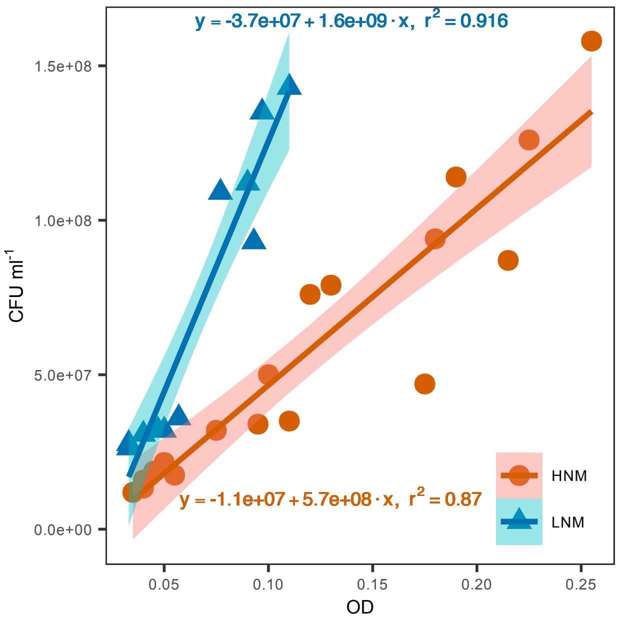

Supplementary figure 2. Colony forming units (CFU) per ml plotted against OD measurements for bacteria in HNM and LNM, data collected from bacteria in exponential phase. The relationship is fitted with a linear model and the shaded area indicates 95% confidence interval. The equations are colour-coded based on treatment.

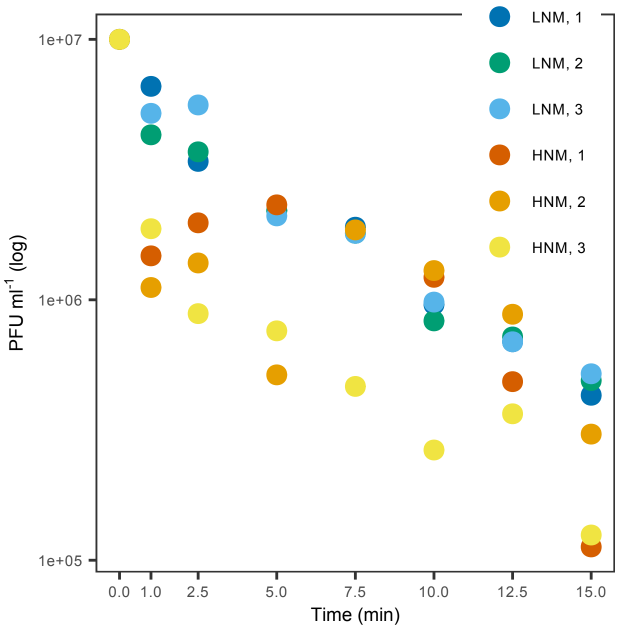

Supplementary figure 3. Adsorption of barba18A phages to BAL341 when mixed at a MOI of 0.1, performed in triplicates in HNM and LNM. Enumeration of the free phages in the mixture were used to calculate plaque forming units. The initial (0 min) concentration of phages were $1x10^7$ PFU ml^{-1}.

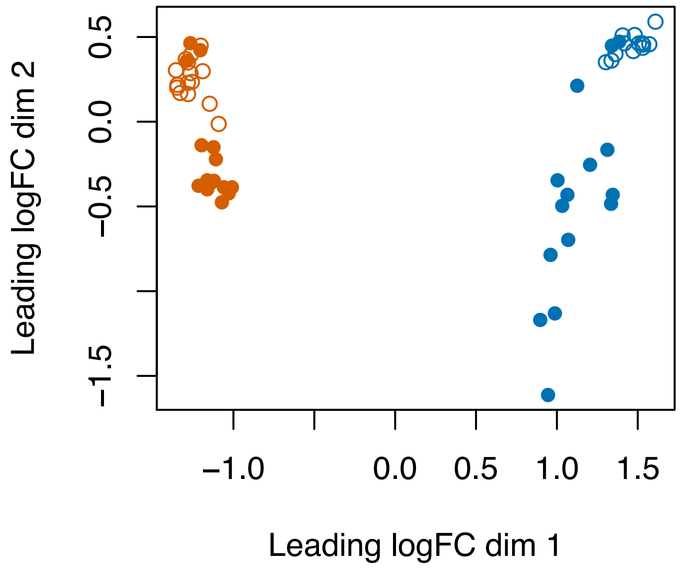

Supplementary figure 4. MDS plot of the mRNA counts associated to bacterial genes in the different samples. Orange circles are samples in the HNM treatment while blue circles are samples in the LNM treatment, which are clearly separated. Closed circles are phage infected bacteria while open circles are non-infected bacteria. For those, phage infected samples at time zero overlap with non-infected samples, while later samples are separated from the uninfected control.

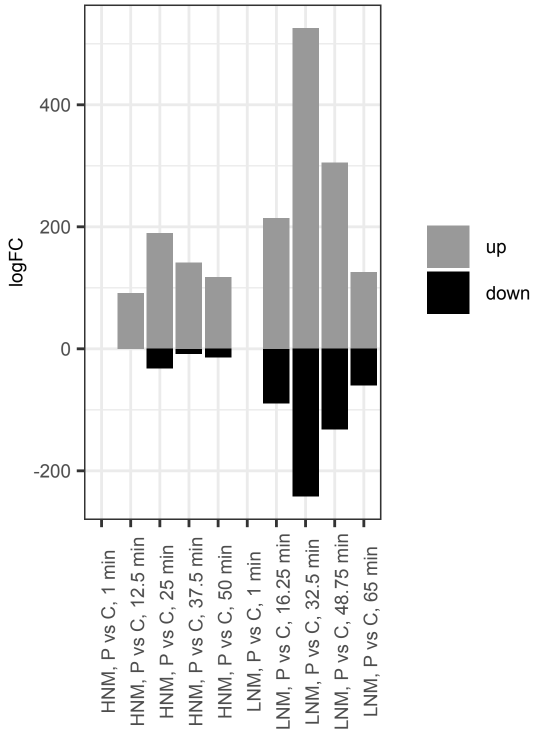

Supplementary figure 5. Overall over- or underexpression (total log2-fold change) of differentially expressed genes for the different samples when comparing phage infected cells (P) to control (C) cells.
